# From Evidence to Engagement: Understanding Online Resistance to Community Water Fluoridation for Public Health Communication—A Qualitative Study

**DOI:** 10.1111/jphd.70061

**Published:** 2026-06-05

**Authors:** Nilesh Torwane, Diep Ha, Ratilal Lalloo, Kristiana Ludlow, Nithin Manchery, Loc Do

**Affiliations:** ^1^ School of Dentistry, the University of Queensland Herston Australia; ^2^ Centre for Health Services Research, the University of Queensland Woolloongabba Australia; ^3^ QIMR Berghofer Brisbane Australia

**Keywords:** community water fluoridation, dental caries prevention, online health misinformation, oral health equity, public health communication

## Abstract

**Objectives:**

This study explored how individuals in an online anti‐CWF community construct and sustain opposition narratives to inform evidence‐based communication and policy engagement.

**Methods:**

A qualitative study was undertaken with adults who self‐identified as not supportive of CWF. Fourteen participants from the United Kingdom, Australia, and the United States of America were recruited via the Fluoride Action Network Facebook page, a prominent global hub for anti‐fluoridation discourse. Semi‐structured interviews were conducted via Zoom between January and July 2025. Data were analyzed using inductive qualitative content analysis. Codes were organized into main, generic, and sub‐categories and quantified by endorsement frequency. Reporting followed SRQR and COREQ guidelines.

**Results:**

Five main categories were identified: (1) Knowledge, Attitudes, Perceptions, and Policy Views; (2) Information Sources and Trust; (3) Reasons for Opposition; (4) Grassroots and Community Actions; and (5) Alternatives and Conditions for Acceptability. Opposition to CWF was shaped by ethical, health, and institutional concerns rather than by scientific disagreement alone. Eight generic categories captured key reasons for opposition, including perceptions of mass medication without consent, health and social harms, institutional distrust, concerns about industrial waste, ethical objections, skepticism about benefits, preference for individualized alternatives, and experiences of professional dismissal. Anti‐fluoride networks and social media were the most trusted sources of information (86%), while trust in mainstream science and health authorities was very low (< 15%).

**Conclusions:**

Opposition to CWF is sustained through network‐mediated information ecosystems and reinforced by behavioral economic mechanisms, including loss aversion, autonomy bias, and default framing effects. Effective public health responses must therefore move beyond evidence dissemination to incorporate network‐aware communication and behavioral insights‐informed strategies that prioritize transparency, public participation, and equitable framing of CWF within broader oral health policy.

## 1 Introduction

Dental caries is the most prevalent chronic condition worldwide, affecting approximately 2.5 billion people and imposing major health, social, and economic burdens [1, 2]. Its impact is greatest among children and socioeconomically disadvantaged populations, where it remains the leading cause of potentially preventable hospital admissions [3, 4, 5].

Community water fluoridation (CWF) is a well‐established public health measure that involves adjusting fluoride concentrations in public water supplies to an optimal level that increases enamel resistance to acid demineralisation [6]. Evidence from longitudinal studies and systematic reviews indicates that CWF reduces caries incidence by 25%–44% in both primary and permanent dentitions [6, 7, 8]. Unlike preventive measures that require individual adherence, such as toothbrushing or regular dental visits, its effectiveness is not dependent on personal compliance [6, 9].

CWF is endorsed internationally, with the World Health Organization recommending it as a safe, cost‐effective preventive measure, and it remains central to oral health policies in Australia, the United States of America, New Zealand, the United Kingdom, and Ireland, among other countries [6, 10]. Nevertheless, implementation remains inconsistent: in Australia, fluoridation is widespread in metropolitan areas but limited in rural and regional communities [11], and some jurisdictions have withdrawn from CWF due to political or community opposition [12]. The consequences of such decisions are well documented. In Calgary, Canada, cessation of CWF in 2011 was followed by a 65% increase in primary‐tooth caries among school‐aged children within 4 years [13]. Similarly, in Byron Shire, New South Wales, repeated rejection of CWF on health, environmental, and ethical grounds has been associated with substantially higher caries rates in children compared with fluoridated regions of the state [14]. These examples illustrate how local policy decisions, often shaped by public sentiment, can diverge from national and international guidance with measurable consequences for oral health.

The role of social media in shaping sentiment around CWF has expanded considerably. Digital platforms facilitate the rapid circulation of health‐related content, enabling advocacy messages to coexist with, or be overshadowed by, misinformation [15, 16, 17, 18]. In several instances, online opposition campaigns targeting public health measures, including CWF, have directly influenced community resistance and, in some cases, policy reversals [14, 19]. Prior research shows that opposition in these spaces is often communicated through narratives that stress uncertainty about health impacts, question the legitimacy of scientific advice, and invoke personal liberty as a counterweight to collective health goals [17, 20]. Network science research demonstrates that online communities tend to cluster around shared beliefs through homophily, with high‐connectivity nodes exerting disproportionate influence on information flow; within such structures, tightly bonded peer groups reinforce social cohesion, amplify in‐group narratives, and systematically limit exposure to differing perspectives [15, 18, 19].

Misinformation in online debates about CWF is especially influential. Research indicates that content with emotional appeal spreads faster and further on social media than factual information and is more resistant to correction [17, 21]. Behavioral economic mechanisms further explain this resistance: loss aversion leads individuals to weight perceived health risks more heavily than equivalent preventive benefits, while social proof, the tendency to adopt the beliefs and behaviors endorsed by one's peer network, confers legitimacy on anti‐CWF claims circulating within these communities [21]. Critically, these online dynamics do not remain confined to digital spaces; they translate into offline behaviors and decision‐making, including community mobilization, council lobbying, and, in some cases, policy reversals [8, 12, 13, 14]. Such dynamics can embed distrust toward health authorities and reduce support for established preventive interventions, such as CWF [22].

Despite recognition of the impact of online opposition to CWF, little is known about the perspectives of individuals who actively engage in these communities. Most prior research has examined population‐level attitudes or broad public opinion [17, 20]. Yet, individuals who consistently contribute to online discussions play a critical role in shaping narratives, framing information, and legitimizing certain viewpoints [16, 23]. Their communication strategies and cited sources often extend beyond personal belief, influencing the tone and direction of broader debate. Understanding these contributors and the rationales underpinning their opposition is therefore essential, as it can inform the design of more targeted communication strategies, help public health authorities anticipate and address familiar narratives, and support policy efforts to sustain CWF in the face of organized resistance [17, 18, 20].

Addressing opposition to CWF requires more than just presenting additional scientific evidence. Communication strategies must engage directly with the values and concerns that drive resistance, while recognizing the influence of digital platforms in sustaining these debates. International advisory bodies, including the World Health Organization, emphasize the importance of transparent and inclusive dialogue with communities to maintain trust and sustain public health programs [6, 10].

This study aimed to examine the perspectives and motivations of individuals actively engaged in online anti‐CWF communities. It explored the narratives, rationales, and communication practices that underpin opposition to CWF in digital spaces. By focusing on the voices of online contributors, this study provides insights that can strengthen evidence‐based health communication and inform practical strategies to sustain and enhance the acceptability of CWF.

## 2 Methods

### 2.1 Study Design

This qualitative study employed one‐on‐one semi‐structured interviews to examine the perspectives and motivations underlying opposition to CWF among individuals active in an online anti‐CWF community. Data were analyzed using inductive qualitative content analysis, an approach that identifies descriptive patterns in data (categories) from participants' accounts and allows for quantification of category prevalence through frequency counting [24]. To strengthen methodological integration, the study was embedded within a mixed‐methods framework, adopting a concurrent triangulation design whereby qualitative insights were complemented by descriptive frequency counts [25]. Reporting followed the Standards for Reporting Qualitative Research (SRQR) [26] and the Consolidated Criteria for Reporting Qualitative Research (COREQ) (Supporting Information) [27].

### 2.2 Participants and Recruitment

Adults (≥ 18 years) who self‐identified as opposed to CWF and could speak English were eligible to participate. Recruitment was conducted via the Fluoride Action Network (FAN) Facebook page, a closed group and the largest anti‐CWF platform on social media, with more than 85,000 members. The FAN page functions as a central hub for anti‐CWF discourse and campaign coordination across the United States of America, Australia, the United Kingdom, New Zealand, and other countries [28]. To access the group, a member of the research team joined the closed Facebook page as a participant observer, after which a study advertisement was posted directly to the page, inviting followers to participate. Interested individuals were directed to a brief Qualtrics‐hosted screening survey (approximately 1 min) that collected demographic information (age, gender, country of residence, and education) and assessed eligibility against the inclusion criteria. Eligible respondents were subsequently contacted directly by the research team via email to arrange an interview. At this stage, participants received a Participant Information Sheet outlining the study aims, confidentiality protections, and their rights as research participants, and written or emailed informed consent was obtained prior to each interview. Participants selected a date and time that were convenient for them, and interviews were conducted via Zoom. Recruitment continued until data saturation was reached, at which point no novel information was identified in successive interviews [29].

### 2.3 Interviewer Training and Reflexivity

Three investigators contributed to the study. All interviews were conducted by NT, a dentist and Dental Public Health researcher who was trained in qualitative interviewing and analysis by KL [30]. The second investigator (NM), a research coordinator trained in qualitative methods, contributed to coding and analytic discussions. KL, a senior research fellow with expertise in health services research and qualitative methods, provided methodological oversight, advising on coding, category development, and analytic consistency. None of the investigators had prior relationships with participants.

Reflexivity was a central consideration. NT's professional background as a dentist and CWF researcher provided contextual knowledge but also risked bias. To mitigate this, NT maintained a neutral and non‐judgemental stance during interviews, focusing on rapport‐building and allowing participants to fully articulate their perspectives without interruption or challenge [31]. Reflexive practices included journaling, analytic memos, and iterative debriefings with KL and NM throughout the study [31]. KL's independent perspective was particularly important for challenging NT's assumptions and ensuring analytic decisions remained grounded in participant accounts. Because interviews were conducted via Zoom, contemporaneous fieldnotes were limited; however, verbatim transcripts, post‐interview reflections, and iterative team discussions supported reflexivity and analytic rigor. Reflexivity and researcher subjectivity were approached in line with established recommendations emphasizing transparency in qualitative analysis [32, 33].

### 2.4 Data Collection

Interviews were conducted online via Zoom between January and July 2025. Each interview lasted between 45 and 90 min (median ~60 min). A semi‐structured guide (Appendix A) explored: socio‐demographic background, core concerns about CWF, perceived health risks and benefits, trusted information sources, institutional trust, and alternative preventive strategies. The interview guide was developed through a review of literature on CWF, health communication, and online health misinformation. It was further refined through discussions with dental public health academics to ensure relevance and content validity. The guide was then pilot‐tested with staff members and PhD candidates from the Population Oral Health Unit at The University of Queensland, with feedback‐informed adjustments made to improve clarity and engagement.

Participants received the guide approximately 1 week in advance and were reminded that they could choose not to answer any questions. With consent, interviews were audio‐video‐recorded. Zoom's Live Transcript AI tool generated transcripts, which were manually reviewed and corrected for verbatim accuracy by NT. Participants were not asked to review transcripts or findings [32, 33], and no financial incentives were offered; participants contributed voluntarily.

### 2.5 Data Management

Each transcript was assigned a unique anonymised code. Identifying information (e.g., names) was removed before analysis. Data were securely stored on The University of Queensland's Research Data Manager (UQRDM) secure server, in accordance with institutional and national standards for data protection.

### 2.6 Data Analysis

Socio‐demographic characteristics collected in the screening survey were summarized descriptively. Transcripts were imported into NVivo 15 for coding and management. While NVivo's AI‐assisted functions supported preliminary pattern detection, all coding and categorization were conducted manually to preserve contextual accuracy. The analysis followed Elo and Kyngäs' (2008) three‐phase inductive content analysis [24].

#### 2.6.1 Preparation Phase

All transcripts were read repeatedly by NT to achieve immersion. The unit of analysis was defined as a sentence or paragraph containing a discrete idea.

#### 2.6.2 Organizing Phase

NT and NM used an inductive approach to code all interview transcripts independently. Related codes were clustered and compared to generate a codebook, which NT developed in consultation with NM, following the hierarchical abstraction process outlined by Elo and Kyngäs [34]. The codebook comprised main categories (first order), generic categories (second order), and, in some instances, subcategories (third order). The semi‐structured interview guide informed the identification of the five main categories: *Participants' Knowledge, Attitudes, Perceptions, and Policy Views*; *Information Sources and Trust*; *Reasons for Opposition to CWF*; *Grassroots and Community Actions*; *and Alternatives to CWF and Conditions for Acceptability*. During the interviews, participants were asked to indicate the *level of trust* (high, moderate, or low) they attributed to various information sources. These self‐reported trust levels were used during coding to classify participants' responses. After the initial codebook was developed, NT and NM cross‐checked a subset of transcripts to compare interpretations, discuss overlaps or divergences, and ensure consistent application of the codebook. This post‐development verification process enhanced analytic rigor and ensured that categories remained grounded in participants' language and meaning. The codebook was iteratively refined through team discussions led by KL, who provided methodological oversight and ensured analytic consistency. Discrepancies were resolved through consensus. Frequency counts were generated for main‐, generic‐, and sub‐categories to indicate the number and proportion of participants endorsing each category at least once. The NVivo matrix function was used to summarize how many participants mentioned each category, particularly across the main categories. A word cloud was generated in Python (using the word‐cloud library) to visualize the most frequently used terms in transcripts, providing an additional lexical overview of the dominant concepts in participants' discourse.

#### 2.6.3 Reporting Phase

Categories were presented as narrative accounts supported with verbatim quotations and frequency counts.

### 2.7 Trustworthiness

Ensuring trustworthiness is essential in qualitative research to demonstrate that the findings are grounded in participants' accounts and produced through a transparent and rigorous analytic process. Trustworthiness was addressed using Lincoln and Guba's criteria [35] of credibility, dependability, confirmability, and transferability. *Credibility* refers to the confidence in the accuracy and truth of the findings and was supported through prolonged engagement with the dataset, coding by two independent researchers, iterative discussions to develop the codebook and the use of verbatim quotes to enhance transparency. *Transferability*—the extent to which findings can be applied to other contexts—was enhanced by providing detailed descriptions of participants' characteristics and contexts, along with illustrative quotes from interviews. *Dependability* reflects the stability and consistency of the research process over time. This was ensured by maintaining a clear audit trail of coding decisions and codebook versions. *Confirmability* refers to the extent to which findings are shaped by participants' accounts rather than researcher bias or assumptions. Confirmability was strengthened by grounding interpretations in participants' narratives rather than in the researcher's preconceptions, supported by reflexive documentation, triangulation among coders, and regular team discussions.

## 3 Results

### 3.1 Participant Characteristics

Fourteen individuals participated in the study. Demographic details are presented in Table 1. Participants were aged between 33 and 76 years (mean = 52.0, SD = 11.9). Most participants were female (*n* = 9; 64%). Educational backgrounds were diverse, spanning from no formal education to postgraduate education (Table 1). Participants self‐identified across a spectrum of worldviews, including liberal/green liberal (*n* = 2; 14%), libertarian (*n* = 2; 14%), conservative/traditional (*n* = 2; 14%), faith‐based (*n* = 1; 7%), scientific/scientific skeptic (*n* = 2; 14%), skeptical/populist (*n* = 1; 7%), and other/unstated (*n* = 4; 29%). Engagement in anti‐CWF activism ranged from less than 5 years (*n* = 3; 21%) to more than 30 years (*n* = 2; 14%), with activities spanning local community mobilization (*n* = 5; 36%), online or social‐media advocacy (*n* = 4; 29%), and policy‐level or lobbying work (*n* = 1; 7%), while several participants reported mixed activities or unspecified activities (*n* = 4; 28%). Geographically, over half of the sample were based in the United Kingdom (*n* = 8; 57%), with four from the United States of America (29%) and two from Australia (14%).

**TABLE 1:** Socio‐demographic and activism characteristics of study participants (*n* = 14).

Characteristics	*n* (%) or Mean (SD)
Gender	Woman: 9 (64%)
	Man: 5 (36%)
Age (years)	Range: 33–76 years; Mean: 52 years (SD = 11.9)
Education	No formal education: 1 (7%)
	High school/O‐level: 3 (21%)
	Vocational/partial degree: 1 (7%)
	Bachelor's degree: 2 (14%)
	Postgraduate degree: 2 (14%)
	Academic/unspecified tertiary: 2 (14%)
	Unstated: 3 (21%)
Self‐identified worldview	Liberal/Green liberal: 2 (14%)
	Libertarian: 2 (14%)
	Conservative/traditional: 2 (14%)
	Faith‐based: 1 (7%)
	Scientific/scientific skeptic: 2 (14%)
	Skeptical/populist: 1 (7%)
	Other/unstated: 4 (29%)
Years opposing CWF	< 5 years: 3 (21%)
	5–10 years: 2 (14%)
	11–20 years: 2 (14%)
	> 20 years: 2 (14%)
	Unstated: 5 (36%)
Activism level	Local/community actions: 5 (36%)
	Online/social media: 4 (29%)
	Policy/lobbying: 1 (7%)
	Mixed activities: 2 (14%)
	Unstated: 2 (14%)
Country	United Kingdom: 8 (57%)
	United States of America: 4 (29%)
	Australia: 2 (14%)

### 3.2 Categories

Five main categories were identified: *Participants' Knowledge, Attitudes, Perceptions, and Policy Views*; *Information Sources and Trust*; *Reasons for Opposition to CWF*; *Grassroots and Community Actions*; and *Alternatives to CWF and Conditions for Acceptability*. Within each main category, generic categories were identified, some of which had subcategories (see Tables 2, 3, 4, 5, 6).

**TABLE 2:** Knowledge, attitudes, perceptions, policy views, and opinions on CWF (*n* = 14).

Generic categories	Subcategories	Participants endorsing *n* (%)	Illustrative quotes
Knowledge and risk perceptions	Fluoridation poses health risks	13 (93%)	“Chronic overexposure can lead to fluorosis, brittle bones, and neurotoxic effects.” (P8)
	Concern about overexposure	11 (79%)	“Older people tell me their bones and hips ache worse since fluoridation.” (P9)
	Uncertainty about dosage control	12 (86%)	“Dosage control in a communal system is imprecise.” (P6)
	Familiar with monitoring policies	5 (36%)	“No one posts results publicly. There's no transparency.” (P9)
Attitudes toward the benefits of CWF	Sees no significant benefit	10 (71%)	“Minimal benefit — especially given modern toothpaste.” (P6)
	Would not accept CWF under any condition	4 (29%)	“No amount of benefit would justify mass medication without consent.” (P6)
Government, policy, and decision‐making	Government motives questionable	12 (86%)	“It's about industry profit, not health.” (P2)
	Skepticism of official explanation	11 (79%)	“I doubt the government's reasons for fluoride.” (P3)
	Lack of real public input	13 (93%)	“Public consultations feel like tokenism.” (P8)
	Awareness of policy mechanisms	3 (21%)	“I've never seen any policy update go to public vote.” (P7)
Acceptance and alternatives to CWF	Acceptable only with consent	6 (43%)	“A model like Switzerland's, where policy changes go to a public referendum, would respect individual choice.” (P8)
	Main reason to accept (if any)	5 (36%)	“Only if truly independent research proved it safe.” (P8)
	Suggested alternatives	12 (86%)	“Oral hygiene education, sugar reduction, topical fluoride (opt‐in), better nutrition, and free dental care.” (P8)
Attitudes toward public health research	Distrusts government health motives	12 (86%)	“Motives for fluoridating water impact my trust in all health initiatives.” (P6)
	Research seen as not open or transparent	9 (64%)	“Too secretive. Research should be open and accessible.” (P9)
	Trusted institutions	6 (43%)	“I trust the local university more than federal health agencies.” (P4)

*Note:* Frequencies indicate the number and percentage of participants who referred to each subcategory at least once; percentages are descriptive, not inferential.

**TABLE 3:** Trust, use, and influence of information sources on CWF among participants (*n* = 14).

Generic categories	Used/influential *n* (%)	High trust *n* (%)	Moderate trust *n* (%)	Low/no trust *n* (%)	Most trusted (named)	Least trusted (named)	Illustrative quotes
Anti‐fluoride NGOs/peer networks	12 (86%)	12 (86%)	2 (14%)	0 (0%)	FAN, Fluoride Free Alliance	—	“I rely heavily on Joy's Telegram channel and the Fluoride Free Alliance UK website…” (P8); “I talk with neighbors who have lived here their whole lives.” (P9)
Community/peers	12 (86%)	13 (93%)	1 (7%)	0 (0%)	Friends, local community	—	“I talked to neighbors who all avoid tap water now.” (P9)
Social media (SM)	11 (79%)	10 (71%)	3 (21%)	1 (8%)	Telegram, Facebook	Mainstream news	“I live on social media—TikTok, Telegram channels, B‐list influencer podcasts—and I pore over conspiracy forums at night.” (P3)
	Use frequency: Daily/Frequent = 9 (64%); Occasionally = 4 (29%); Rare/None = 1 (7%)						“I check activist pages daily.” (P6); “I see posts but rarely comment.” (P5); “I prefer community meetings over online.” (P4)
	Perspective changed by SM: 4 (29%); Family/friends influence via SM: 8 (57%)						“Seeing the adverse event post made me dig deeper.” (P3); “I talked to neighbors who all avoid tap water now.” (P9)
Selective peer‐reviewed literature	8 (57%)	—	—	—	—	—	“When I encounter pro‐fluoridation claims … I trace them back to the original studies, scrutinizing methodologies …” (P8)
Health professionals	4 (29%)	2 (14%)	5 (36%)	7 (50%)	—	Health authorities	“I spoke once to our town nurse. She said, ‘Guidelines say it's safe.’ But then she looked down and whispered …” (P9)
Mainstream science	2 (14%)	1 (7%)	4 (29%)	9 (64%)	—	Government sources	“When I encounter pro‐fluoridation claims, I trace them back to the original studies … I'm wary — so much of the science is industry‐funded or only shows benefits under very specific conditions.” (P8)
Mainstream media/journals	2 (14%)	—	—	—	—	—	“I avoid big news channels — I feel they hide who pays them.” (P9)

*Note:* Frequency counts indicate the number and percentage of participants who referenced each source at least once. Percentages are descriptive and not inferential.

**TABLE 4:** Reasons behind opposition to CWF (*n* = 14).

Generic categories	Subcategories	Participants endorsing *n* (%)	Illustrative quotes
Mass medication and lack of consent	Mass medication without consent	13 (93%)	“We wouldn't medicate an entire community against their will with a prescription drug. Yet here we are.” (P6) “Compulsory fluoridation is a violation of individual autonomy—mass medication without explicit consent.” (P8)
	Lack of informed choice	12 (86%)	“The public has no say or choice. We are forced to accept medicated water regardless of consent.” (P5) “It feels like someone is putting medicine into our water without asking us.” (P9)
	Forced exposure/violation of rights	12 (86%)	“Compulsory fluoridation is a violation of individual autonomy—mass medication without explicit consent.” (P8)
Industrial waste disposal	Disposal of industrial by‐products	12 (86%)	“Fluoridation is a method for industry to dispose of toxic by‐products under the guise of public health.” (P2) “Hexa is an industrial waste by‐product of fertilizer manufacture and a biocide.” (P4)
	Neurotoxin and lack of dosage control	10 (71%)	“Fluoride is a neurotoxin at certain doses, dosage control in a communal system is imprecise, and it offers no choice to individuals.” (P6)
Health and social harms	General health risks	13 (93%)	“Chronic overexposure can lead to dental fluorosis, brittle bones, and neurotoxic effects. Environmentally, fluoridated effluent harms aquatic ecosystems.” (P8)
	Harms to vulnerable groups	10 (71%)	“Older people tell me their bones and hips ache worse since fluoridation. I worry about children's growing brains.” (P9)
	Dental fluorosis	9 (64%)	“My son got white lines on his teeth. The dentist called it fluorosis. I felt my heart break, thinking I'd poisoned him.” (P9)
	Neurotoxicity and cognitive effects	11 (79%)	“Neurotoxicity in developing brains, endocrine disruption, bone fragility, and environmental impacts on aquatic ecosystems.” (P6)
Institutional distrust	Government and regulatory distrust	13 (93%)	“Governments are private corporations masquerading as public bodies. It's all controlled by the same people who control the money.” (P2) “My trust is shattered when I see research funded by companies selling fluoride chemicals.” (P6)
	Perceived collusion/lack of transparency	13 (93%)	“Overwhelming distrust of government, water authorities, and perceived corporate capture of regulatory bodies.” (Multiple participants)
Dismissal by professionals	Professional/medical dismissal	9 (64%)	“Doctors are too indoctrinated to question what they've been taught.” (P2) “Health professionals seem reluctant to have their indoctrination questioned.” (P5)
	Doubt among professionals	7 (50%)	“Our town nurse said, ‘Guidelines say it's safe,’ but then whispered, ‘I worry about my child's teeth.’” (P9)
Skepticism of benefit	Doubt about effectiveness	10 (71%)	“The benefit is minimal compared to the risks, especially given modern dental care.” (P6) “Dental fluorosis is not cosmetic—it's the first visible sign of fluoride intoxication.” (P4)
Preference for alternatives	Oral hygiene education	13 (93%)	“Comprehensive oral‐hygiene education in schools can instil good brushing habits early.” (P8) “Teach children proper brushing and offer free dental check‐ups for low‐income families.” (P9)
	Sugar reduction policies	8 (57%)	“Enforce stricter sugar‐reduction policies in processed foods and school meals.” (P8)
	Opt‐in/topical fluoride	10 (71%)	“Use fluoride varnish only when parents ask and understand it. Let families decide.” (P9)
	Free dental care/community programmes	9 (64%)	“Expand access to affordable or free preventive dentistry for underserved populations.” (P8)
Equity and ethics	Lack of fairness/forced exposure	12 (86%)	“We are forced to accept medicated water regardless of consent. No one asks for our views.” (P5)
	Drug definition concerns	8 (57%)	“Fluoride fits the definition of a drug in many regulatory frameworks, yet it's mass‐dosed through water without individual choice.” (P6)

*Note:* Frequency counts indicate the number and percentage of participants who endorsed each category at least once. Percentages are descriptive, not inferential.

**TABLE 5:** Grassroots and community actions reported by participants to oppose CWF (*n* = 14).

Generic categories	Participants endorsing *n* (%)	Illustrative quotes
Founding or leading local groups	7 (50%)	“I co‐founded a local chapter of the Fluoride Action Network here in Queensland, organizing town‐hall meetings, distributing research summaries, and liaising with concerned parents and environmental groups.” (P6)
Social media forums and peer activist networks	11 (79%)	“I live on social media—TikTok, Telegram channels, B‐list influencer podcasts—and I pore over conspiracy forums at night.” (P3) “I rely heavily on Joy's Telegram channel and the Fluoride Free Alliance UK website for alerts on spills, regulatory notices, and community actions.” (P8) “I check activist pages daily for leads on new studies, upcoming council votes, or citizen science initiatives measuring local fluoride levels.” (P6)
Community petitions and letter‐writing campaigns	10 (71%)	“For about ten years, I've written letters—sometimes my hand shook—to the water board. I've prayed with neighbors outside council meetings holding signs that say, ‘Let Us Choose.’” (P9) “I share petitions in local Facebook groups, leave comments on city‐council livestreams, and once helped a friend draft an email to her township board.” (P3)
Organizing or attending public meetings	9 (64%)	“We have organized community town halls and handed out flyers at local events.” (P8) “I've stood up at council meetings, even if it made me nervous.” (P5)
Neighborhood and peer mobilization	10 (71%)	“I try to talk to my neighbors and get them involved. Sometimes we meet up to discuss next steps.” (P9) “A lot of us coordinate via local Telegram or WhatsApp groups.” (P8)
Online activism and sustained digital engagement	12 (86%)	“I've been speaking out online and in my neighborhood for about two years. I share petitions, leave comments on council livestreams, and help others draft letters.” (P3) “We organize action alerts via Telegram channels whenever there's a council vote or news story.” (P8)
Supporting and educating peers	8 (57%)	“I help new members learn how to write to the council and where to find reliable information.” (P6) “Sometimes, I just listen to others' stories and encourage them to speak up.” (P10)
Persistence despite setbacks	9 (64%)	“Even if nothing changes, I feel it's important to keep going. We can't let them think everyone agrees.” (P9) “It gets tiring, but someone has to keep trying.” (P6)

*Note:* Percentages are descriptive and not inferential.

**TABLE 6:** Participant‐suggested alternatives and acceptability conditions for CWF (*n* = 14).

Generic categories	Subcategories	Participants endorsing *n* (%)	Illustrative quotes
Preferred alternatives to CWF	Oral hygiene education (brushing/flossing)	9 (64%)	“Teach children proper brushing and flossing early in school with real toothbrushes.” (P9)
	Topical fluoride (varnish/toothpaste), opt‐in	8 (57%)	“Use fluoride varnish only when parents ask and fully understand it. Let each family decide.” (P9)
	Sugar reduction policies	7 (50%)	“Enforce stricter sugar‐reduction policies in processed foods and school meals to tackle the root cause of decay.” (P8)
	Free or affordable dental check‐ups	6 (43%)	“Offer free dental check‐ups for kids whose families cannot pay.” (P9)
	Community‐based dental health programmes	6 (43%)	“Comprehensive oral‐hygiene education delivered by community health workers—especially in linguistically diverse areas.” (P8)
	Diet and nutrition programs	5 (36%)	“Provide good meals at community centres.” (P9)
	Filtered or bottled water options	5 (36%)	“Everyone should see test results every week, posted in our town centre, so we can choose what to drink.” (P9)
	Restrict fluoride use to high‐risk individuals	4 (29%)	“Reserve fluoride therapies—like topical varnishes or toothpaste—for high‐risk individuals who opt in, rather than dosing an entire population.” (P8)
Conditions for acceptability of CWF	Genuine public consent (referenda or binding votes)	6 (43%)	“A model like Switzerland's, where policy changes go to a public referendum, would respect individual choice.” (P8)
	Independent peer‐reviewed safety evidence	6 (43%)	“Truly independent peer‐reviewed research earns my trust.” (P8)
	Transparent monitoring and public reporting	5 (36%)	“Everyone should see test results every week, posted in our town centre, so we can choose what to drink.” (P9)
	Strict opt‐out or choice available	5 (36%)	“Let each family decide.” (P9)
	Targeted use for high‐risk populations only	4 (29%)	“Reserve fluoride therapies for high‐risk individuals who opt in.” (P8)
	None; would not accept under any circumstances	4 (29%)	“No amount of benefit would justify mass medication without consent.” (P6)

*Note:* Frequency counts indicate the number and percentage of participants who endorsed each alternative or criterion at least once; percentages are descriptive, not inferential.

#### 3.2.1 Participants' Knowledge, Attitudes, Perceptions, and Policy Views

This main category comprised five generic categories—*Knowledge and Risk Perceptions, Attitudes Toward the Benefits of CWF, Government, Policy, and Decision‐Making, Acceptance and Alternatives to CWF, and Attitudes Toward Public Health Research*—and 16 subcategories (Table 2). Most participants (*n* = 13; 93%) perceived CWF as posing health risks, with (*n* = 11; 79%) expressing specific concerns about chronic overexposure and (*n* = 12; 86%) highlighting uncertainty about dosage control. Only a minority of participants (*n* = 5; 36%) demonstrated familiarity with local monitoring or reporting mechanisms. Participants frequently referred to issues such as “chronic overexposure,” “imprecise dosage,” and a “lack of transparency in public reporting,” underscoring perceptions of inadequate oversight. Regarding attitudes and risk perceptions, most participants (*n* = 10; 71%) believed that CWF offers little or no additional benefit relative to contemporary dental care practices, while four participants (*n* = 4; 29%) stated that they would not accept CWF under any circumstances.

Policy—and governance‐related views further reflected deep skepticism toward institutional motives. The majority of participants (*n* = 12; 86%) questioned government intentions, and (*n* = 12; 79%) doubted the official rationale for fluoridation policies. Only three participants (*n* = 3; 21%) were aware of any mechanisms for meaningful public consultation or policy updates. Broader public health trust also appeared to be affected: 12 participants (86%) reported that perceptions of CWF negatively influenced their trust in other health initiatives, nine (64%) described fluoridation research as “too secretive,” and six (43%) expressed higher trust in local academic institutions than in federal agencies.

#### 3.2.2 Information Sources and Trust

Six *generic categories* related to participants' sources of information and levels of trust *were identified* (Table 3). Substantial variation was observed in how participants accessed and evaluated information about CWF. *Anti‐fluoride NGOs and peer networks* were both widely used and highly trusted (*n* = 12, 86%), functioning as primary information and coordination channels. *Community and peer groups* were described as highly influential (*n* = 12, 86%) and highly trusted (*n* = 13, 93%), often serving as local verification networks for information circulating online. *Social media platforms* were the principal source of CWF information (*n* = 11, 79%), with participants expressing high trust in content shared within these networks (*n* = 10, 71%) and reporting daily or frequent engagement (*n* = 9, 64%). Several participants cross‐referenced information found on Telegram or Facebook with activist forums to verify or contextualize claims (*n* = 9, 64%).

In contrast, *health professionals* were viewed with high trust by only a few participants (*n* = 2, 14%), and mainstream scientific institutions by just one participant (*n* = 1, 7%). Reliance on selective peer‐reviewed or independent studies perceived as free from institutional bias was reported by more than half of the participants (*n* = 8, 57%). At the same time, *mainstream media or government‐linked scientific outputs* were reportedly used by only two participants (14%).

#### 3.2.3 Reasons for Opposition to CWF

Eight generic categories and 20 subcategories underlying opposition to CWF were identified (Table 4). Participants frequently framed fluoridation as a form of *mass medication without consent* (*n =* 13; 93%), emphasizing the violation of individual autonomy and lack of informed choice. This included accounts of *forced exposure* and the perception that consent was impossible when fluoridation was applied at the community level.

Concerns about *health and social harms* were also prominent (*n =* 13; 93%). Participants described risks ranging from general health impacts (e.g., brittle bones, endocrine disruption) to specific conditions (e.g., dental fluorosis and neurotoxicity), as well as harms to vulnerable groups and environmental impacts. *Institutional distrust* (*n =* 13; 93%) was evident in strong skepticism toward government and regulatory agencies. Participants expressed beliefs about collusion between authorities and industry, a lack of transparency in decision‐making, and resistance to freedom of information requests, which they viewed as evidence of systemic capture.

Alongside opposition, participants expressed a *preference for alternatives* to CWF (*n =* 13; 93%). These included preventive approaches such as oral hygiene education, sugar‐reduction policies, topical fluoride use on an opt‐in basis, and expanding access to free or affordable dental care. Such proposals were often framed as more ethical, targeted, and respectful of choice.

Other categories included industrial *waste disposal* (*n =* 12; 86%), with participants arguing that fluoridation served as a means to dispose of toxic by‐products; *equity and ethics* (*n =* 12; 86%), particularly around fairness, forced exposure, and whether fluoride should be classed as a drug; *skepticism of benefit* (*n =* 10; 71%), with participants questioning whether any health gains justified the risks; and *professional or medical dismissal* (*n =* 9; 64%), where participants reported feeling ignored or belittled by health professionals when raising concerns. These eight categories highlight the multifaceted nature of opposition to CWF, combining ethical, health, institutional, and policy dimensions.

#### 3.2.4 Grassroots and Community Actions

The analysis identified seven *generic categories* and eight *subcategories* describing participants' grassroots and community‐based activities opposing CWF (Table 5). These included *founding or leading opposition groups* (*n* = 7; 50%), participation in *social media forums and activist networks* (*n* = 11; 79%), and *community petitioning or letter‐writing campaigns* (*n* = 10; 71%). Most participants (*n* = 10; 71%) described coordinating community efforts through local or online networks, particularly via Telegram, Facebook, and WhatsApp, to mobilize attendance at public hearings and share information on regulatory developments. Public engagement was another prominent activity. Most participants reported *organizing or attending public meetings* (*n* = 9; 64%) and *mobilizing neighbors or local communities* (*n* = 10; 71%), reflecting ongoing collective efforts to influence local policy and raise awareness.

*Digital activism* played a central role, with 12 (86%) reporting sustained online engagement, including posting petitions, sharing scientific reports, and coordinating “action alerts” before council votes. Participants also described *supporting and educating peers* (*n* = 8; 57%), particularly newcomers to advocacy, by teaching communication strategies and providing informational resources. Collectively, these findings underscore that CWF opposition extends beyond online rhetoric to include structured, sustained, and community‐organized activism, often blending local action with digital coordination.

#### 3.2.5 Alternatives to CWF and Conditions for Acceptability

The analysis identified two *generic categories* and 12 *subcategories* related to participants' views on acceptable alternatives and conditions for CWF (Table 6). Participants expressed a strong preference for individualized and preventive approaches to oral health over community‐wide fluoridation. The most frequently proposed *preferred alternatives to CWF* included oral hygiene education (*n* = 9; 64%), opt‐in or topical fluoride use (*n* = 8; 57%), and sugar reduction policies (*n* = 7; 50%). Additional suggestions encompassed free or affordable dental check‐ups (*n* = 6; 43%), community‐based dental health programmes (*n* = 6; 43%), nutrition‐focused initiatives (*n* = 5; 36%), filtered or bottled water options (36%), and targeted fluoride use for high‐risk individuals (*n* = 4; 29%).

When asked about *Conditions for acceptability of CWF*, participants most commonly cited the requirement for genuine public consent through referenda or binding votes (*n* = 6; 43%), independent peer‐reviewed safety evidence (*n* = 6; 43%), transparent public monitoring and reporting of fluoride levels (*n* = 5; 36%), and the presence of strict opt‐out provisions (*n* = 5; 36%). A minority of participants (*n* = 4; 29%) stated that CWF would be unacceptable under any circumstances, viewing it as incompatible with individual autonomy and informed consent. These findings reflect a consistent emphasis on personal choice, scientific independence, and procedural transparency as key determinants of acceptability.

### 3.3 Dominant Language and Key Terms

**FIGURE 1:**
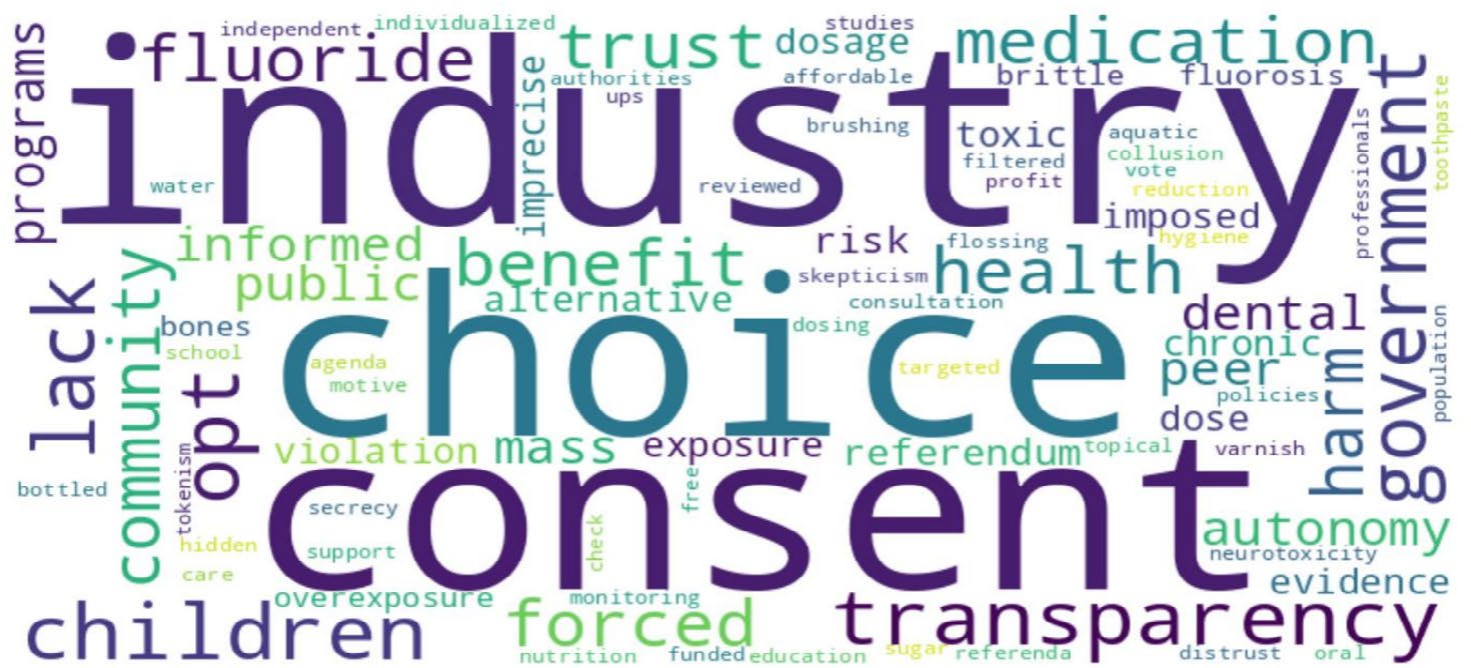
Word cloud of dominant terms used by Participants about CWF. The size of each term reflects its relative frequency in the interview dataset. [Color figure can be viewed at wileyonlinelibrary.com]

The most frequently used words and phrases by participants are illustrated in Figure 1. Terms such as “consent,” “risk,” “forced,” “harm,” “choice,” “toxic,” and “autonomy” were most prominent, reflecting the central concepts of perceived individual rights, health risks, and opposition to mandatory interventions.

## 4 Discussion

This study examined how individuals in an online community explained and sustained their opposition to CWF. The findings show that opposition is not simply the result of a limited understanding of scientific evidence but is instead a complex, values‐driven stance. Ethical concerns, institutional distrust, and perceived health and social harms emerged as central categories in participants' opposition to CWF. These positions were articulated by individuals from three countries, consistent with earlier studies showing that anti‐fluoridation sentiment arises across populations and is not confined to particular socioeconomic groups [14, 36, 37]. While some research suggests that specific rationales may be emphasized more strongly within particular groups (e.g., liberty and consent arguments in libertarian or conservative communities, or scientific critiques among more highly educated participants) [36], overall opposition has been documented across the socio‐economic spectrum.

It is important to note, however, that all participants were members of a single online community united by choice homophily, the tendency of individuals to selectively affiliate with others who share their beliefs and values. This shared network membership likely reinforced and amplified oppositional views, irrespective of participants' geographic or demographic differences. A substantial body of evidence demonstrates that social networks are among the most powerful determinants of health beliefs and health‐related decision‐making, often exerting greater influence than formal health information or clinical advice [16, 17, 38]. Within such networks, peer validation, shared narratives, and coordinated activism mutually reinforce one another, creating conditions in which opposition to CWF becomes socially normative and self‐sustaining.

A key finding was the widespread framing of CWF as “mass medication without consent.” This objection, also prominent in prior research, shifts the debate from the health sciences to the domain of ethics and autonomy [36, 39]. For many participants, the issue was not whether CWF prevents dental caries, but whether it violates the principle of individual choice. This helps explain the preference for alternatives such as topical fluoride or oral hygiene programs, which are perceived as voluntary measures [5, 36].

Participants expressed a profound distrust of government and public health institutions. Fluoridation was described as emblematic of broader failures of transparency and accountability, a view aligned with earlier findings that opposition to CWF reflects deep‐seated institutional mistrust [14, 36, 40]. When official sources were distrusted, participants said that they turned to peer networks, advocacy groups, or social media communities. These sources provided affirmation, identity, and solidarity, but also perpetuated misinformation. Like other public health debates, in‐group validation reinforced existing beliefs and made them resistant to correction [16, 17, 20, 38].

Participants also raised perceived health risks such as neurotoxicity and claims that fluoride is an “industrial waste by‐product.” These assertions diverge from the position of international health authorities [12, 13, 14] but were framed as evidence of deliberate concealment by officials. Such claims align with broader research on conspiracy beliefs, in which risk perception is shaped not by the weight of scientific evidence but by suspicion of authority and narratives of hidden truth [17, 37, 41]. Some participants expressed conditional trust in local or independent academic institutions, suggesting that credibility is more closely linked to perceived independence than to expertise alone [36, 40]. These accounts collectively indicate that participants' trust hierarchies were shaped less by scientific authority than by perceived independence and peer credibility.

The role of digital media was central. Many participants reported engaging in activism, including signing petitions, lobbying, and participating in coordinated online campaigns. They Social media platforms enabled the rapid spread of emotionally charged narratives, often overshadowing factual information. Prior studies confirm that misinformation in these environments spreads faster than corrective messages, with emotionally resonant content being particularly influential [15, 19, 23, 37, 42]. Once established, these narratives are difficult to dislodge and can exert substantial influence on local decision‐making processes.

The implications of opposition to CWF for oral health are significant. Evidence from Canada, Australia, and other jurisdictions demonstrates that discontinuation of CWF is followed by measurable increases in dental caries, disproportionately affecting children and disadvantaged populations [8, 9, 13, 40, 41]. Comprehensive reviews and meta‐analyses confirm that CWF is one of the most effective and equitable strategies for preventing tooth decay [3, 4, 5, 6, 7, 42]. Policy reversals driven by misinformation therefore risk undermining population‐level gains in oral health and widening inequities [40, 41, 43].

### 4.1 Study Implications

This study contributes new knowledge by providing in‐depth qualitative insights into how and why individuals actively oppose CWF, an area that has been under‐researched. By analyzing opposition narratives, the study shows that concerns extend beyond factual disputes about safety or efficacy to include issues of autonomy, consent, institutional distrust, and ethical framing. These findings complement existing quantitative evidence [8, 9, 10] on the effectiveness of CWF by illuminating the social and communicative dynamics that drive resistance.

The implications for policy and practice are threefold. First, understanding opposition through the lens of values (e.g., liberty, fairness, and transparency) underscores the need for communication strategies that address both ethical and scientific arguments. Second, identifying the central role of peer networks and alternative information spaces highlights the urgency of developing proactive digital engagement and prebunking (proactive misinformation inoculation) strategies to counter misinformation where it spreads. Third, documenting the breadth of rationales across diverse demographic groups suggests that tailored, context‐sensitive approaches are required rather than one‐size‐fits‐all messaging.

Based on these implications, we propose a set of practical recommendations across three priority domains: transparency and ethics, community and digital engagement, and equity, education, and framing to strengthen CWF communication and policy. Several examples are drawn from evaluated public health strategies in other domains, providing evidence that these approaches can be effective when adapted to CWF. Practical recommendations developed from the study findings are summarized in Table 7. While many originate from analogous domains such as vaccination, nutrition fortification policies, and COVID‐19 risk communication campaigns, these approaches have demonstrated effectiveness in building public trust, improving engagement, and countering misinformation; therefore, they provide relevant insights for adaptation to CWF. Together, these recommendations highlight how insights from this study can inform more effective communication strategies, rebuild public trust, and support evidence‐based decision‐making to sustain and expand CWF as a core component of oral health equity policy.

**TABLE 7:** Practical recommendations for improving CWF communication and policy, with illustrative evidence‐based examples.

Priority area	Recommendation	Example/application (with evidence)
Transparency & ethics	Publish real‐time fluoride levels, water‐testing results, and operational updates in accessible formats.	Public health dashboards (e.g., COVID‐19 dashboards) increased public trust through real‐time transparency [44].
	Acknowledge consent issues; compare CWF to accepted, equity‐oriented population interventions (e.g., iodised salt, folic acid fortification).	Framing fortification as fairness/equity improved acceptability in nutrition policy [45].
Community & digital engagement	Go beyond surveys to include citizen panels, deliberative forums, and co‐produced resources for open dialogue.	Deliberative dialogue in health promotion increased legitimacy and co‐design with communities [46].
	Use prebunking and values‐based storytelling; maintain a consistent presence on social media to counter false claims early.	Psychological inoculation reduced uptake of misinformation [34].
	Create mechanisms for resident Q&A with timely responses from health officials (e.g., moderated forums, hotlines).	Behavioral‐science‐informed campaigns during COVID‐19 used interactive channels to improve accessibility and reduce the spread of misinformation [47].
	Partner with trusted local messengers (health professionals, teachers, community leaders) to deliver consistent pro‐CWF messages.	Trusted‐messenger interventions improved vaccine uptake among minority populations, demonstrating the value of local credible voices [48].
Equity, education & framing	Present CWF as part of a broader oral‐health strategy (sugar reduction, school programs, preventive services).	WHO oral‐health policy supports integrated, equity‐focused approaches; school programs complement CWF [49].
	Emphasize population‐level effectiveness and equity benefits.	Multilevel analyses in Australia show that CWF is effective and reduces disparities [8].
	Highlight adult oral‐health gains to broaden relevance beyond childhood.	Fluoridated drinking water is associated with fewer caries in Australian adults [9].
	Use relatable narratives and local stories to increase message uptake and trust.	Narrative communication enhances persuasion and recall in health contexts [50].
	Involve young people as advocates through peer‐led, school/university campaigns.	Youth ambassador and inclusion approaches improve engagement and peer‐to‐peer knowledge transfer in oral health [51].

### 4.2 Limitations

This study is subject to several limitations. Recruitment from a single closed anti‐CWF Facebook group, the FAN page, likely over‐represented strongly oppositional views, and the findings reflect the experiences and beliefs of individuals within this specific online social network rather than online opposition to CWF more broadly. As a qualitative study, the findings are not intended to be generalized; rather, they offer an in‐depth understanding of how participation in this particular online community shaped participants' beliefs and opposition narratives around CWF. Restricting the sample to English‐speaking participants excluded perspectives from culturally and linguistically diverse communities. Online interviews, while efficient and flexible, may lack the rapport and depth that face‐to‐face settings offer. Future studies should incorporate neutral and supportive perspectives, investigate how political and service contexts shape trust, and evaluate whether strategies such as transparency and deliberative engagement can shift public views and policy outcomes. Longitudinal designs could further explore whether such approaches influence both attitudes and oral health outcomes.

## 5 Conclusion

This qualitative study provides insight into how individuals' experiences within an online social network shaped their beliefs about CWF. Among this group of participants, opposition appeared to be driven not primarily by engagement with scientific evidence, but by ethical objections, institutional distrust, and values rooted in autonomy and identity perspectives, likely reinforced through shared network membership and choice homophily. This opposition is further sustained through network‐level dynamics, including information cascades, homophily, and the outsized influence of high‐connectivity hubs such as the FAN Facebook page, which amplify oppositional narratives and limit exposure to corrective information. Behavioral economic mechanisms, including loss aversion, autonomy bias, and default framing effects, help explain the persistence of these beliefs even in the presence of established scientific consensus. While these findings are not generalisable, they offer a nuanced understanding of the lived experiences and belief‐forming processes of individuals actively engaged in an online anti‐CWF community. These insights underscore the limitations of evidence‐only communication approaches and suggest that public health efforts may benefit from drawing on network science and behavioral economics, building transparency, engaging communities through trusted network nodes, and framing CWF as part of a broader oral health equity strategy to better engage with values‐driven resistance and support the preventive benefits of this long‐established intervention.

## Funding

N.T. is supported by a University of Queensland Earmarked PhD Scholarship under L.D.’s NHMRC Ideas #2024439.

## Ethics Statement

Ethical approval was obtained from The University of Queensland Human Research Ethics Committee (2024/HE000315). All participants received a Participant Information Sheet outlining study aims, confidentiality protections, and their rights as research participants. Written or emailed informed consent was obtained before each interview. Participants could decline any question or withdraw at any time without consequence.

## Consent

All participants provided informed consent before participation in the study.

## Conflicts of Interest

The authors declare no conflicts of interest.

## Supporting information


**Appendix A** Structure of the interview guide.

**Data S1:** COREQ (COnsolidated criteria for REporting Qualitative research) Checklist.

## Data Availability

The data that support the findings of this study are available on request from the corresponding author. The data are not publicly available due to privacy or ethical restrictions.
