# Supplementary material for: From Evidence to Engagement: Understanding Online Resistance to Community Water Fluoridation for Public Health Communication—A Qualitative Study
Source: J Public Health Dent. 2026 Jun 5;86(3):284–300. doi: 10.1111/jphd.70061 (PMC13547111; doi:10.1111/jphd.70061)
Supplement: Supplementary file 2 [file JPHD-86-284-s001.docx]

**Appendix A**

**Structure of the interview guide**

**Preamble**

1. The participant will get the consent letter and an overview of the study via email before the interview from the researcher. Information about age, gender, education, libertarian philosophy, and attitudes toward science will all be included in the questionnaire. Before the interview, the participants must read a research overview and the study's objectives. After agreeing to participate in the study and for the interview to be recorded, the participants will be requested to submit a signed or emailed letter of consent.
2. Participants will also be allowed to fill out the interview questions (and return them via email) if they feel uncomfortable with the process.

**“The Interview Script and Study Questionnaire”**

Hello (name),

My name is (Researcher Name, Position and Affiliation).

Thank you for agreeing to participate in today's interview. I value your participation in my study and your openness to meeting with me today. This study intends to shed light on the reasons for and issues with opposition to community water fluoridation (CWF). The project will examine public concerns about water fluoridation and allow participants to offer alternatives to fluoridating public water supplies.

You may withdraw from the interview and research at any time, and all information you provide will be kept confidential. In addition to taking notes throughout the conversation, I will also record its audio. Please confirm verbal approval so we can document it.

Thank you.

First, I will discuss today's procedure, and then we will proceed.

This one-time one-on-one conversation will take 45 to 50 minutes. During the interview, I will ask you questions based on a plan we developed. I want your perspective on community water fluoridation in general and population/public health research.

Do you have any questions regarding the interview procedure?

Let's get started.

**First, I would like to know about yourself**

- What is your education level:
- What ideology do you believe in? (Please select the appropriate option)

a. Libertarian	b. Liberal	c. Conservative	d. Communitarian	e. Centrist	f. Any other	g. None	h. Prefer Not To Say

- What is your attitude towards science?

a. Positive	b. Negative	c. Neutral	d. Sceptical

- Do you have engagement in water-fluoridation opposition lobby activities?
- How long have you been engaged in these activities?

**Now, I would like to understand your knowledge about community water fluoridation.**

- What is community water fluoridation?

- What thoughts come to mind when I say “community water fluoridation”?

- What is your understanding of the processes of water fluoridation? (e.g., how is fluoride added to water?)

- Can you tell me any personal experiences (negative or positive consequences) or stories about water fluoridation? From your own life, family, friends, or other community members?
- Where do you get information about water fluoridation practices?
- How often do you use social media to gather information about community water fluoridation?
- Remember the last time you encountered a post or discussion about the safety and effectiveness of community water fluoridation on social media. Did it change or influence your perspective in any way?
- Which organisations or types of content creators do you find most trustworthy when it comes to information about fluoride on social media, and why?
- What happens when you see articles, papers, media, or other sources of information on community water fluoridation that contradict your opinion? How do you react?
- Was there a situation where you consulted family members, friends, or others about community water fluoridation? If yes, can you elaborate on that conversation?
- Have you ever discussed fluoride with a physician, dentist, or other health care professionals? If yes, can you describe the outcome of this discussion?

**Next, I would like to know your opinions regarding community water fluoridation.**

- What resources or past experiences led to your current perspective or belief about water fluoridation?
- Tell me about how you began expressing your opposition to fluoridation.
- In your opinion, what are the possible concerns or risks of community water fluoridation practices? (e.g., environmental, health), What is the most critical risk?
- How familiar are you with fluoride dosage in community water systems?
- Do you have any concerns about overexposure to fluoride through community water fluoridation? What health effects do you think this could cause? Can you provide an example?
- What is your opinion on how community water fluoridation dosage levels should be monitored and reported to the public?
- What do you think are the main reasons why the government implements fluoridation in community water supplies?
- Do you have any doubts or scepticism about the government's reasons for promoting fluoride in drinking water? If so, what are they?
- How is the decision to fluoridate public water made in your community? Who are the key stakeholders involved in this decision?
- Are you aware of any current policies regarding the monitoring and adjustment of fluoride levels in public water systems in your area?
- Are there any recent or proposed changes to fluoridation policies in your community? What prompted these changes?
- Are any mechanisms available for public input or opposition regarding community water fluoridation?
- Are there any benefits to community water fluoridation?
- What is the most important benefit?
- Under what circumstances might community water fluoridation be acceptable, if any?

Do you think there are any alternatives to water fluoridation in preventing oral diseases? If yes, what possible alternatives to water fluoridation could you suggest to the government to benefit the community's oral health?

**Finally, I would like to know your opinions on Public Health Research**

- As a public member, how do you feel about health research, in general?

- How accessible do you feel health information is to you?

- How do the government's motives for fluoridating water impact your trust in other public health initiatives?"

- How do you feel about the openness of health research information? (i.e., not secretive, honest, and accessible to you)

- Whom do you think should or should not be involved in the research process of public health interventions? Why?

- Are there any organisations that you trust more than others? Less than others? Have you interacted with any of these institutions? (e.g., the public health community, the dental community, universities or any others)

**Thank you very much for taking the time out of your schedule to meet with me today. If you have any questions or concerns, please don’t hesitate to contact me at (email address)**
